# Risk and predictors of dyssynchrony cardiomyopathy in left bundle branch block with preserved left ventricular ejection fraction

**DOI:** 10.1002/clc.23467

**Published:** 2020-09-17

**Authors:** Sunita Sharma, Harsh V. Barot, Andrew D. Schwartzman, Sarju Ganatra, Sachin P. Shah, David M. Venesy, Richard D. Patten

**Affiliations:** ^1^ From the Division of Cardiovascular Medicine Lahey Hospital and Medical Center Burlington Massachusetts USA

**Keywords:** cardiomyopathy, dyssynchrony, heart failure, left bundle branch block

## Abstract

**Background:**

Left bundle branch block (LBBB) and left ventricular (LV) dyssynchrony likely contribute to progressive systolic dysfunction. The evaluation of newly recognized LBBB includes screening for structural heart abnormalities and coronary artery disease (CAD). In patients whose LV ejection fraction (EF) is preserved during initial testing, the incidence of subsequent cardiomyopathy is not firmly established.

**Hypothesis:**

The risk of developing LV systolic dysfunction among LBBB patients with preserved LVEF is high enough to warrant serial imaging.

**Methods:**

We screened records of 1000 consecutive patients with LBBB from our ECG database and identified subjects with an initially preserved LVEF (≥45%) without clinically relevant CAD or other cause for cardiomyopathy. Baseline imaging, clinical data, and follow‐up imaging were recorded to determine the risk of subsequent LV systolic dysfunction (LVEF ≤40%).

**Results:**

(Data are mean + SD) 784 subjects were excluded, the majority for CAD or depressed LVEF upon initial imaging. Of the remaining 216, 37 (17%) developed a decline in LVEF(≤40%) over a mean follow‐up of 55 ± 31 months; 94% of these patients had a baseline LVEF≤60% and LV end systolic diameter (ESD) ≥ 2.9 cm indicating that these measures may be useful to define which patients warrant longitudinal follow‐up. The negative predictive value of a LVEF>60% and LVESD <2.9 cm was 98%.

**Conclusions:**

Seventeen percent of patients with LBBB and initial preserved LVEF develop dyssynchrony cardiomyopathy. We believe the risk of developing dyssynchrony cardiomyopathy is high enough to warrant serial assessment of LV systolic function in this high‐risk population.

**Abbreviations**
BNP: brain natriuretic peptide
CAD: coronary artery disease
CRT: cardiac resynchronization therapy
EDD: end diastolic dimension
EF: ejection fraction
ESD: end systolic dimension
LBBB: left bundle branch block
LV: left ventricular

## 1 INTRODUCTION

The prevalence of left bundle branch block (LBBB) in the general population ranges from approximately 0.1% to 1.0%, the incidence increasing with age.^1^, ^2^, ^3^, ^4^, ^5^ LBBB is strongly associated with structural heart and/or coronary artery disease.^3^, ^5^, ^6^, ^7^, ^8^ Patients with a newly recognized LBBB are at increased risk of cardiovascular events including heart failure, myocardial infarction, and sudden death.^3^, ^6^, ^8^ The evaluation of patients with incidental, newly recognized LBBB therefore, necessitates assessments for structural heart disease and coronary artery disease (CAD) in appropriate candidates, an approach that is supported by current guidelines.^9^ Clinical and experimental data support that dyssynchronous left ventricular (LV) contraction (ie, early septal activation with delayed lateral wall contraction) itself may lead to a decline in LV systolic function.^10^, ^11^, ^12^, ^13^ In patients with LBBB and a reduced LV ejection fraction (LVEF), cardiac resynchronization therapy (CRT) improves survival, and reduces heart failure hospitalizations.^14^, ^15^, ^16^ Among patients treated with CRT, reports describe “super‐responders” whereby LVEF normalizes with resolution of heart failure symptoms.^17^, ^18^ These observations have led to the notion that LBBB with resultant dyssynchrony may play a causative role in the development or progression of LV systolic dysfunction. This putative syndrome is now commonly designated “dyssynchrony cardiomyopathy” or “LBBB‐associated cardiomyopathy.”^19^, ^20^ Among patients with LBBB and preserved LV systolic function upon initial imaging, the likelihood of developing a dyssynchrony‐induced cardiomyopathy, and whether such patients require longitudinal follow‐up is not well established. The purpose of our study was to determine the risk of and explore predictors of developing a cardiomyopathy by examining a population of patients with LBBB, an initially preserved LVEF, without clinically relevant coronary artery disease, or other potential cause of LV systolic dysfunction.

## 2 METHODS

### 2.1 Patient population

This study was approved by the Institutional investigational review board. We obtained subjects by screening our ECG database from September 2011 to September 2012 for all studies in which LBBB was included in the official interpretation. This time period represents the earliest computerized tracings for review in our database to assure as long a follow‐up period as possible. The ECG was analyzed for evidence of a left bundle branch block using conventional criteria that included native QRS duration ≥120 ms, broad R waves in leads I, aVL, V5, or V6, and absent q waves in leads I, V5, and V6.^21^ Among those with a confirmed LBBB, we reviewed all prior available ECGs to establish the earliest date in which the LBBB was identified for a given subject. This served as time point zero or the start of the follow‐up period.

Patients were included if they demonstrated an initially preserved LVEF prospectively defined as ≥45% by any imaging modality obtained after the earliest identified LBBB tracing with at least one follow‐up assessment of LV function. Transthoracic echocardiography was the imaging method used for 205 out of 216 subjects (95%). A LVEF of 45% was chosen as the lower limit because dyssynchrony alone acutely lowers the EF in patients with rate‐related LBBB and no other structural heart abnormalities.^11^, ^22^ Moreover, 2D echocardiography has been shown to underestimate LVEF in subjects with and without LBBB compared with quantitative 3D modalities.^23^, ^24^ To reduce selection bias inherent in the need for repeated, follow‐up imaging, we included patients with only one assessment of LV function if their ejection fraction was preserved (≥45%) and measured more than 1 year after the LBBB was identified. Patients whose LBBB was noted to be intermittent or rate‐related were excluded. Subjects were also excluded if they had no assessment of LV function. Subjects who demonstrated an initial LVEF of <45% or whose medical history noted any other potential cause of cardiomyopathy or LV systolic dysfunction were also excluded as were patients with severe aortic or mitral valve disease. Subjects with clinically relevant coronary artery disease were excluded. This was defined as a history of a myocardial infarction, prior coronary angiogram demonstrating a ≥ 75% stenosis of a major epicardial coronary artery, percutaneous coronary intervention on a major epicardial vessel or previous coronary artery bypass surgery. Patients were excluded, if they had a pacemaker or were censored at the time of pacemaker implant though they remained in the study population, if pacemaker interrogations were available and demonstrated less than 10% right ventricular pacing. The follow‐up period was defined as the date starting with the earliest ECG demonstrating a LBBB through the date of the latest echocardiogram.

### 2.2 Assessment of variables

Medical records were reviewed whereby clinical data were recorded at the time of initial diagnosis including age, gender, comorbid conditions (eg, hypertension, diabetes mellitus), and laboratory data (BUN, creatinine, hemoglobin, BNP/NT‐proBNP when available). The initial and follow‐up EKGs were reviewed for underlying rhythm, QRS axis, and duration. We obtained echocardiographic measurements (LVEF, end diastolic dimension, and end systolic dimension) from echocardiographic reports. Chamber dimensions were only available for those patients whose initial LVEFs were assessed by echocardiography and whose reports were available for review (N = 152 for patients with retained preserved LVEF—Group 1; N = 31 for patients whose LVEF deteriorated—Group 2). Our institution follows the chamber quantification guidelines for these measures.^25^ End diastolic and end systolic dimensions were not available in patients whose LVEF assessment was performed by another modality (eg, ventriculogram, gated myocardial perfusion imaging, MRI).

### 2.3 Statistical analysis

Two groups were compared: patients whose LVEF remained preserved (≥45) upon subsequent imaging—Group 1; and those who exhibited a decline in LVEF (≤40%) on subsequent imaging—Group 2. Continuous variables were expressed as mean ± SD. Comparisons were made with the student *t* test. Chi square analysis was used to compare differences in the frequencies of categorical variables between the two groups. Differences were considered statistically significant if the *P*‐value was less than .05. Odds ratio, sensitivity, specificity, and negative predictive values were calculated using standard formulas. We utilized Kaplan‐Meyer analysis to chart the time course of the development of LV systolic dysfunction among those patients who developed a cardiomyopathy during the follow‐up period. The Symstat software (version 13.2) was used for these analyses.

## 3 RESULTS

**FIGURE 1:**
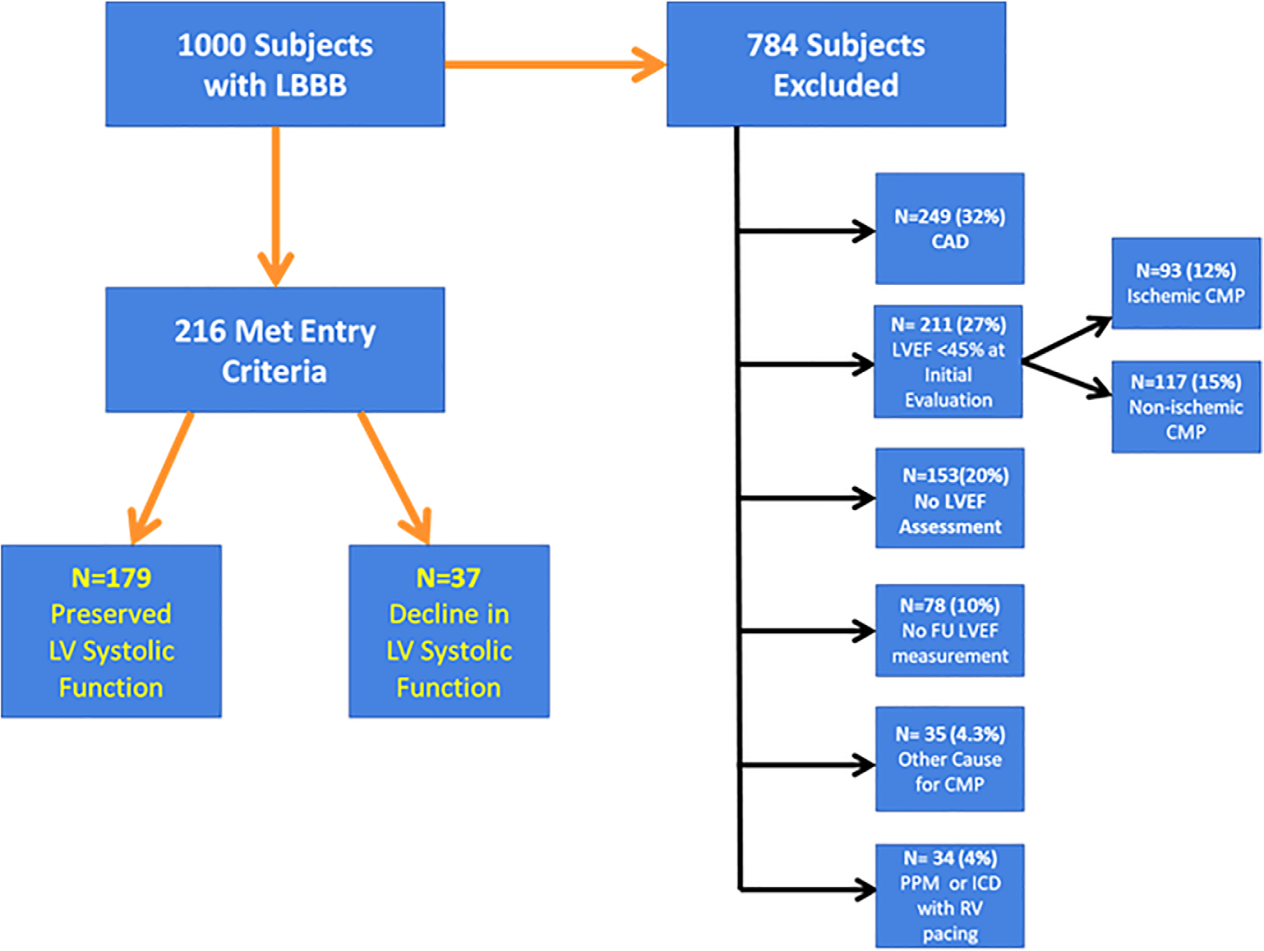
**Study population‐reasons for exclusion.** Flow chart depicting the subjects screened for this analysis with excluded patients and reason for exclusion shown on the right. 24 patients were excluded (not shown) because the LBBB was found to be intermittent (n = 22) or the ECG did not meet criteria for LBBB (n = 2)

We reviewed a total of 1000 consecutive subjects within our ECG database, between September 2011 and September 2012 with a LBBB. Of these, 786 were excluded for reasons shown in Figure 1. 24 were excluded because the ECG did not meet accepted criteria for LBBB (n = 2) or the LBBB was found to be intermittent or rate related upon review of subsequent tracings (n = 22). Clinically relevant coronary artery disease was the most common reason for exclusion (N = 249). An additional 93 patients were excluded because of a known ischemic cardiomyopathy at the time the LBBB was identified whereas 117 patients demonstrated a diminished LVEF (<45%) on their earliest imaging study without a clear etiology. An additional 35 subjects had a CMP due to an identifiable cause, such as, hypertrophic cardiomyopathy, chemotherapy‐induced cardiomyopathy, sarcoidosis, or amyloid. 153 subjects were excluded because they lacked any assessment of LV function; 78 patients were excluded due to absence of a follow‐up assessment of LV function. 34 patients were excluded because of RV pacing. The remaining 216 patients met the inclusion criteria and were included in this analysis. Among these, 179 patients exhibited continued preserved LV systolic function (Group 1) over a follow‐up period of 66.9 ± 37.0 months whereas 37 patients (17%) exhibited deterioration of LV systolic function (Group 2: LVEF ≤40%) during a mean follow‐up period of 55.1 ± 30.5 months with the median time to a decline in LVEF of 48 months. Table 1 demonstrates the demographic and clinical variables pertaining to these groups. We observed a female predominance (70%) among those patients who maintained a preserved LVEF compared with 51% females among those who developed a decline in their LVEF. The incidence of cardiovascular risk factors, such as, diabetes mellitus, hypertension, and dyslipidemia were statistically similar between the two groups. Body mass index (not shown), baseline renal function, and hemoglobin were also similar between these groups. Among those with available BNP levels, Group 2 (N = 24) demonstrated significantly higher BNP levels than Group 1 (N = 91) though the standard deviations were wide.

**TABLE 1:** Baseline clinical characteristics

Baseline characteristics	Preserved LVEF (≥45%;Group 1; N = 179)	Decline in LVEF (≤40%; Group 2; N = 37)
**Follow‐up (months)**	66.9 (12‐194)	55.1 (12‐125)^a^
**Age (years)**	72.6 ± 11.6	73.1 ± 10.3
**Female gender**	126 (70%)	19 (51%)^a^
**Hypertension**	139 (78%)	26 (70%)
**Diabetes mellitus**	32 (18%)	7 (19%)
**BUN (mg/dl)**	18.5 ± 10.1	19.4 ± 8.3
**Creatinine (mg/dl)**	0.98 ± 0.34	1.01 ± 0.32
**Hgb (mg/dl)**	13.2 ± 1.5	13.3 ± 2.0
**HgbA1C**	6.0 ± 1.1	6.0 ± 0.7
**BNP ng/ml**	213 ± 238 (n = 93)	487 ± 438^b^ (n = 28)

Abbreviations: BUN, blood urea nitrogen; BNP, brain natriuretic peptide; HgbA1c, glycosylated hemoglobin; Hgb, hemoglobin; LVEF, left ventricular ejection fraction.

^a^ *P* < 0.05.

^b^ *P* < 0.001.

**FIGURE 2:**
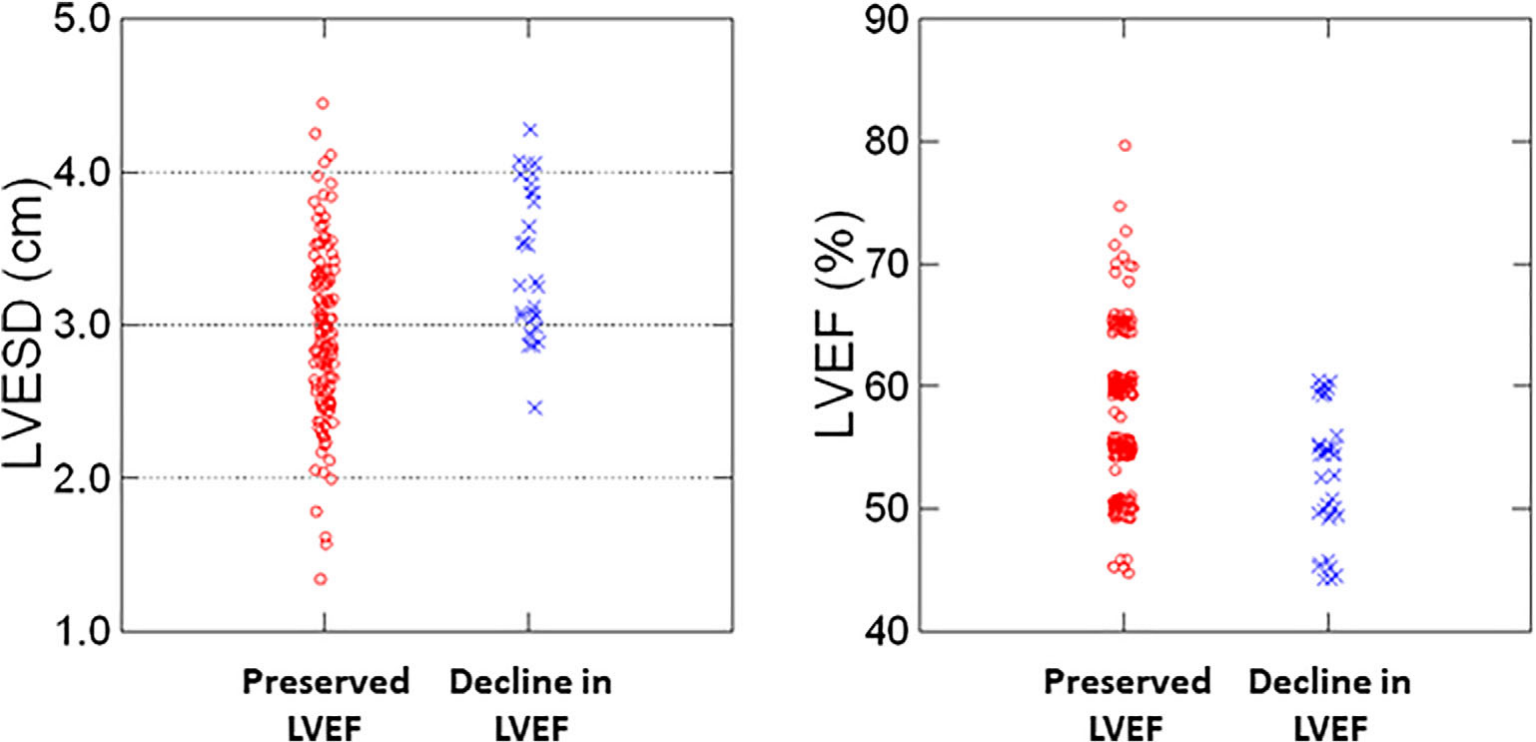
**Scatter plots of *baseline* left ventricular end systolic diameter (ESD) and left ventricular ejection fraction (LVEF).** Although there is great overlap between the groups, the vast majority (94%) of patients who developed a cardiomyopathy (decline in LVEF, Group 2) demonstrated an initial LVESD of ≥2.9 cm whereas slightly more than half of the preserved LVEF group had an initial LVESD ≥2.9 cm. No patients who developed a cardiomyopathy had an initial LVEF above 60%. In patients with an LVESD of ≥2.9 cm and an LVEF ≤60%, the relative risk of developing a cardiomyopathy was 18.4. The negative predictive value for an LVESD <2.9 cm and LVEF >60% was 98%

Table 2 shows the ECG and echocardiographic data. A smaller percentage of patients in Group 2 were in sinus rhythm on the earliest LBBB ECG (84% vs 96% in Group 1, *P* < .01). The baseline mean QRS durations and QRS axes were similar. The follow‐up QRS durations in the decline in EF group, Group 2, were slightly and significantly higher than those in the preserved LVEF group, Group 1. The initial assessments of LVEF differed significantly with a statistically lower mean LVEF in those with subsequent deterioration of LV systolic function vs those who maintained a preserved LVEF. Coinciding with the LVEF measures, the initial LVEDD and LVESD measurements were higher in the group whose LVEF declined. Data pertaining to baseline LVEFs and LVESDs are shown graphically as scatter plots in Figure 2. Although there is a great deal of overlap between Group 1 and Group 2, no patient in Group 2 (decline in LVEF) exhibited an initial EF of more than 60% or an initial LVESD of less than 2.5 cm. In fact, among patients with an LVESD≥2.9 cm, the odds ratio for developing a decline in LVEF was 14.5 (95% confidence interval 3.3‐62.9). Combining the variables of LVESD≥2.9 cm and LVEF ≤60%, the odds ratio for developing a decline in LVEF was 18.4 (95% Confidence interval 4.2‐79.8). The sensitivity of these combined parameters in identifying those who subsequently developed dyssynchrony cardiomyopathy is 94% with a specificity of 56%. Accordingly, the negative predictive value of having an LVESD of <2.9 cm and an LVEF of >60% was 98%.

**TABLE 2:** ECG and echocardiographic data

ECG, echocardiographic data	Preserved LVEF (≥45%; Group 1; N = 179)	Decline in LVEF (≤40%; Group 2; N = 37)
**Sinus rhythm (N [%])**	171 (96%)	31 (84%)^a^
**Initial QRS duration (ms)**	141 ± 14	142 ± 15
**QRS axis**	−17 ± 34	−14 ± 31
**Initial LVEF (%)**	57.7 ± 6.3	53.1 ± 5.3^b^
**Initial LV end diastolic diameter (cm)**	4.45 ± 0.61 (n = 152)	4.93 ± 0.59^b^ (n = 31)
**Initial LV end systolic diameter (cm)**	2.90 ± 0.54 (n = 152)	3.43 ± 0.49^b^ (n = 31)
**F/U QRS duration (ms)**	142 ± 13	146 ± 14^b^
**F/U LVEF (%)**	56.5 ± 6.2	32.3 ± 5.7^b^

Abbreviations: F/U, follow‐up; LVEF, left ventricular ejection fraction.

^a^ *P* < 0.01.

^b^ *P* < 0.001.

**FIGURE 3:**
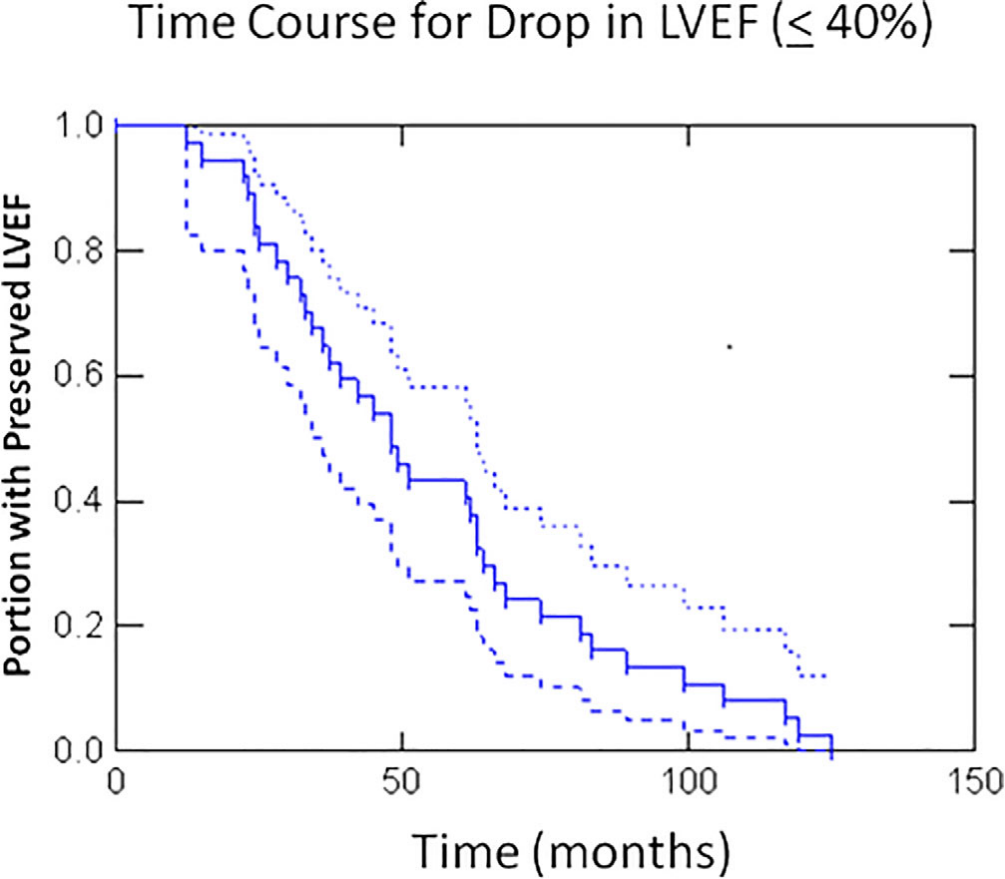
**Time course for the development of dyssynchrony cardiomyopathy.** Kaplan‐Meier curve of the time course for the decline in LV systolic function among the cardiomyopathy group (Group 2). The dashed lines represent the 95% confidence intervals. The mean follow‐up period was 55 ± 31 months with the median time to develop a LVEF ≤40% being 48 months. All patients in Group 2 developed a cardiomyopathy within 125 months after the first identification of a LBBB

Figure 3 shows the time course of the decline in LVEF after the earliest identifiable LBBB ECG. Kaplan‐Meier analysis of this group revealed a median time of 48 months with 1/3 of the patients exhibiting a decline in LVEF within the first 24 months.

## 4 DISCUSSION

Significant interest has been placed on dyssynchrony cardiomyopathy^20^ following the recognition of “super responders” who demonstrate normalization of LV systolic function following CRT lending great support to the hypothesis that dyssynchrony itself plays a causative role in progressive LV systolic dysfunction in patients with LBBB.^17^, ^18^ However, the risk of developing dyssynchrony cardiomyopathy among patients with LBBB and initial preserved LVEF is not well established. Indeed, the purpose of the present study was to address this question and identify predictive variables for the development of dyssynchrony cardiomyopathy.

Because of the need for sequential imaging to document an initially preserved LVEF with subsequent deterioration, retrospective analyses are wrought with selection bias. To reduce this bias, we included patients with a single assessment of LV systolic function if that subject's LVEF was preserved and the assessment occurred more than 1 year following the earliest LBBB ECG. By identifying the earliest time at which the LBBB was identified we were able to achieve a relatively long follow‐up duration that extended to more than 10 years with a mean of more than 5 years. Henceforth, we report here that 17% of patients with LBBB and an initial preserved LVEF develop subsequent LV systolic dysfunction (LVEF≤40%) indicative of dyssynchrony cardiomyopathy.

Other groups have tried to answer this question using varied approaches. In a landmark study, Vaillant et al.^18^ examined a CRT database to identify those with LBBB‐induced cardiomyopathy, who responded to CRT with normalization of systolic function. Out of 375 subjects, they identified six who met their criteria giving an incidence of 1.6%. To be included in this data set; however, subjects had a known LBBB with preserved LVEF for at least 5 years that have may have underestimated the true incidence of dyssynchrony cardiomyopathy. Indeed, within our database, the median time to the development of LV systolic dysfunction was 4 years indicating that many comparable subjects in the study by Vaillaint et al.^18^ would have been excluded. Angheloiu et al.^26^ reported that 16% of 49 patients with an initially preserved LVEF and LBBB developed LV systolic dysfunction over 4 years. A more recent study by Sze et al.^27^ utilized an echocardiography database and identified 94 cases with LBBB and an initial preserved LVEF. 36% of these subjects developed a subsequent decline in LVEF (defined as ≤45%) over a median follow‐up of 4 years. Although they reported a higher percentage than that observed within our dataset, the LVEFs in a significant portion of their cardiomyopathy population were between 40% and 45%.

An important strength of our study lies in the identification of the earliest LBBB‐ECG in each subject, labeling this as time zero. The time for the development of dyssynchrony cardiomyopathy among subjects in our population would therefore be applicable to how such patients are identified and followed in clinical practice. All 37 patients within this cohort developed LV systolic dysfunction within 10.5 years. Importantly, 117 patients from our original 1000 subject database had a reduced LVEF on their earliest imaging assessment with no other identifiable cause for their cardiomyopathy. It is possible, therefore, that many if not the majority of these patients had LV systolic dysfunction driven by dyssynchrony.

A correlate of dyssynchrony cardiomyopathy is the decline in LVEF observed with chronic right ventricular (RV) pacing. RV pacing was recognized as a potential causative factor in heart failure progression in the DAVID trial^28^ and in patients with normal ejection fraction in the MOST trial.^29^ The risk of developing dyssynchrony cardiomyopathy observed within our dataset is not dissimilar from RV pacing‐induced cardiomyopathy reported in approximately 10% to 20% of patients.^30^, ^31^, ^32^

Within the present study, there were no specific clinical or ECG variables that predicted which patients would subsequently develop a reduction in LVEF. However, some potentially important findings were noted within the echocardiographic data. For instance, none of the patients who developed a cardiomyopathy had a LVEF greater than 60% at their initial imaging evaluation. Moreover, within the cardiomyopathy cohort, all of the patients had an LVESD ≥2.5 cm and the majority of patients (94%) had an LVESD ≥2.9 cm on their initial echocardiogram. These data support those subjects with LBBB, who develop a decline in LV systolic function display features of early adverse LV remodeling as indicated by greater chamber size and slightly lower initial LVEF. To our knowledge, this is the first such report whereby these readily available echocardiographic measures aid in identifying those with LBBB at greatest risk for developing dyssynchrony cardiomyopathy.

It is well established that a depressed LVEF is an independent risk factor for cardiovascular morbidity and mortality.^33^ A study from the Mayo clinic^34^ demonstrated that patients with mildly to moderately reduced LVEF and LBBB have significantly worse survival than matched patients without conduction disease. Moreover, in a separate study by Sze et al., once significant LV systolic dysfunction is established, medical therapy may have less impact on reverse remodeling in subjects with LBBB compared with those with nonspecific conduction delay or a narrow QRS complex.^35^ Hence, the early identification of patients in whom a cardiomyopathy is evolving is crucial to reducing morbidity and mortality. Because of the relatively high risk of developing LV systolic dysfunction in patients with LBBB, our data support longitudinal follow‐up and serial imaging in this population. Indeed, given that many patients may be asymptomatic as their cardiomyopathy evolves, identifying such patients in this early phase of dyssynchrony cardiomyopathy may have life‐saving implications as early CRT may prevent worsening LV systolic function and delay the development of overt heart failure.^17^, ^36^

To date, there are no guideline statements to address how patients with LBBB are managed longitudinally. Based on our data and that from others,^18^, ^26^, ^27^ the formation of guidelines outlining the longitudinal management of patients with LBBB is strongly supported. Accordingly, we propose that patients with a LBBB and an initial preserved LVEF undergo annual clinical assessment coupled with imaging to evaluate LV systolic function.

## 5 LIMITATIONS

Since inclusion within our database required serial assessments of LVEF after the diagnosis of LBBB, the possibility of selection bias remains. Moreover, despite screening 1000 subjects with LBBB, over 75% were excluded from the data reducing our sample size. We were also unable to include morbidity or mortality assessments within our population as these data could not be obtained reliably. Moreover, medication data were only inconsistently available; it is possible; therefore, that differences in background medical therapy (eg, beta‐adrenergic blocker or ACE‐inhibitor therapy) may have influenced our findings. Finally, echocardiographic data were extracted from written reports and were not subjected to core‐lab verification.

## 6 CONCLUSIONS

Dyssynchrony cardiomyopathy occurs in 17% of patients with LBBB and preserved LVEF on initial assessment over a mean follow‐up period of 55 months. An initial LVEF between 45% and 60% and an LVESD ≥2.9 cm are associated with much greater risk of developing LV systolic dysfunction among patients with LBBB. Our findings support that patients with LBBB and a preserved LVEF undergo serial imaging and clinical assessments.

## CONFLICT OF INTEREST

The authors declare no potential conflict of interests.

## Data Availability

Data available from the authors on request.
